# Traditional medicine utilisation and maternal complications during antenatal care among women in Bulilima, Plumtree, Zimbabwe

**DOI:** 10.1186/s40748-021-00130-w

**Published:** 2021-02-09

**Authors:** Nicholas Mudonhi, Wilfred Njabulo Nunu

**Affiliations:** 1 Department of Environmental Science and Health, Faculty of Applied Sciences, National University of Science and Technology, Corner Gwanda Road and Cecil Avenue, Ascot, P O Box AC 939, Bulawayo, Zimbabwe; 2 Scientific Agriculture and Environment Development Institute, Bulawayo, Zimbabwe

**Keywords:** Traditional medicine, Maternal complication, Antenatal care, Bulilima, Plumtree, Zimbabwe

## Abstract

**Background:**

As part of the expectation enshrined in the Sustainable Development Goals, countries are expected to ensure maternal health outcomes are improved. It follows that under ideal circumstances, pregnant women should deliver safely without complications, neonatal, and maternal mortality. This paper analyses the relationship between traditional medicine utilisation and maternal complications during antenatal care among women in Bulilima, Plumtree, Zimbabwe.

**Methods:**

A quantitative cross-sectional survey was conducted on 185 randomly selected women who responded to a pre-tested semi-structured questionnaire. The Fisher’s Exact Test and the Test of Proportions were used to probe the relationship between traditional medicine utilisation and the prevalence of maternal complications using STATA SE Version 13.

**Results:**

Complications were reported by (51) 29% of the women who were under study. The proportion of women who developed complications was higher in those that did not use traditional medicine as compared to those that used traditional medicine (30 and 26% respectively). In a generalised assessment, women who did not use traditional medicine contributed a significantly higher proportion of complications as compared to those that utilised traditional medicine.

**Conclusion:**

This study found a significant relationship between the utilisation of traditional medicines and lesser chances of experiencing maternal complications. Significantly higher prevalence of maternal complications was observed in women who did not use traditional medicine compared to those that did. There is, therefore, a need to investigate further the constituents or active ingredients in this traditional medicine. This study provides a window of opportunity for fully recognising and integrating traditional medicine into Modern Health Systems. It can be argued that traditional medicine utilisation could be a viable alternative to modern medicine, particularly in resource-poor settings where access to modern medicine is seriously constrained.

## Introduction

One of the desirable results among women is to bring forth a healthy bouncing child [1]. Therefore, in addressing unfinished agendas of the Millennium Development Goals (MDGs) and strengthening recently agreed Sustainable Development Goal (SDG) of maternal mortality reduction of 7.5% per year between 2016 and 2030 [2], there is need to look at traditional medicine carefully uses during antenatal care. Globally, strategies have been put in place to curb maternal mortality and complications among pregnant women, but none of them takes into account traditional medicine utilisation and complications [3]. Antenatal care model and birth preparedness and complication readiness (BPCR) promote birth planning by improving health-seeking behaviour to ensure timely and appropriate care during pregnancy, labour, delivery, and the postnatal period [4]. Maternal complications are unpredictable and can be fatal within a short space of time [5]. For instance, a severe postpartum haemorrhage can lead to death in less than 2 h, and the unborn foetus may succumb much earlier [5, 6]. Each year in Africa, an estimated quarter of a million women die of problems related to pregnancy, while nearly half die around the time of childbirth, and during the first week after birth [7]. Several complications, such as bleeding obstructed labour, eclampsia, and infections, make up the most significant causes of mothers’ deaths, accounting for two-thirds of maternal mortality in Sub-Saharan Africa [8]. The costs of dealing with some complications such as a caesarean section in Sub-Saharan African (SSA) countries can bankrupt a family [7].

Without exempting Zimbabwe, maternal and neonatal mortality rates in much of SSA are unacceptably high [9]. An estimated 3000 women die every year in Zimbabwe during childbirth, and at least 1.23% of Gross Domestic Product (GDP) is lost annually due to maternal complications [10]. Zimbabwe is ranked among the top countries with high maternal mortality and pregnancy-related complications, as indicated by the Maternal Mortality Rates (MMRs) of 790/100000, 570/ 100,000 and 443/100000 in years 2008, 2010 and 2015 respectively [10]. Despite the adoption of the “Equity in Health” policy soon after independence, there are still inequities as there are unmet needs in as far as rural women are concerned, rendering them more susceptible to maternal mortality and pregnancy-related complications [11]. In a study conducted in Harare, it was revealed that 52% of woman utilising traditional medicines were significantly associated with null parity and null gravidity [12].

Studies have suggested that over 80% of the Zimbabwean rural populace, including those residing in Bulilima utilise traditional medicines for different ailments and purposes [13]. Women do utilise these traditional medicines during pregnancy, and there is a dearth of knowledge that addresses/explores the complications that arise due to the utilisation of traditional medicine during pregnancy [14]. The majority of women utilise traditional medicines to prevent unforeseen complications that could occur during pregnancy and delivery [15]. Debates have erupted that question the safety of these traditional medicines has not been sufficiently explored [16]. This study, therefore, sought to explore the association between traditional medicine utilisation and maternal complications during antenatal care among women in Bulilima, Plumtree, Zimbabwe.

## Methods

### Study area

**Fig. 1:**
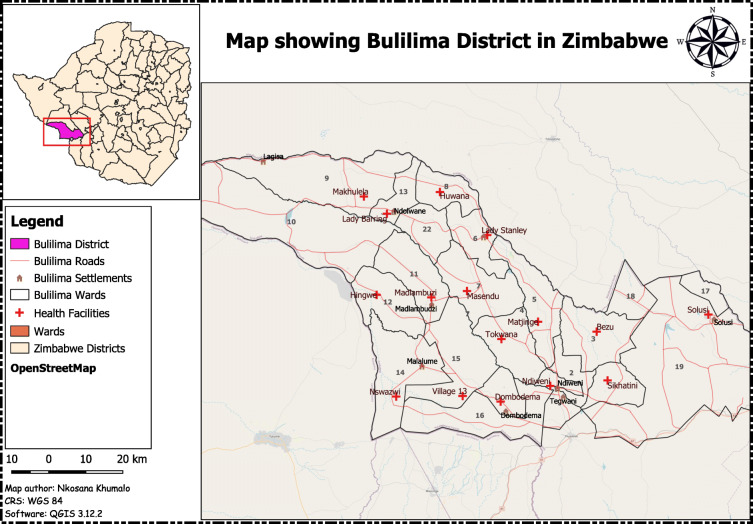
Map showing secondary schools in Bulawayo, Zimbabwe

Bulilima District is located in Matabeleland South province and bordering with Botswana to the west in Region Five, which is prone to severe drought [17]. It has sixteen clinics, twenty-two wards, and has a population of 94,361, with 54% females with Kalanga and Ndebele being the dominant tribes [18]. The district has twenty-two wards, one main referral hospital with sixteen clinics that usually refer pregnant women with complications to the district hospital, and has an average household size of five [18]. The majority of people speak Kalanga and Ndebele as their dominant language. The average distance that women walk to the nearest clinic is estimated to be 5–10 km. The study area is illustrated in Fig. 1.

### Study design

A quantitative cross-sectional survey was conducted on women who had delivered in Bulilima District. This study design enabled the researchers to explore complications that were experienced by women during their pregnancy and delivery and also determine whether or not they used traditional medicine. This study design was cost-effective and enabled data to be collected over a short space of time without having to follow up on women (conducting a study in a snapshot) [19].

### Population and sampling

The study targeted 586 women who delivered either at home or clinic from January–December 2019 in Bulilima District. The sample size was determined using a sample size calculator on EPI INFO using a Confidence Interval of 95% with a width of confidence of 5% and an expected value of the attribute of 50%. This estimation gave a sample size of 185. The respondents were then randomly selected through the use of random numbers. Those selected were accessed, consent sought from them and a questionnaire administered. This sample size was sufficient to conduct statistical analysis and make inferences that could be generalised to the whole population of interest in Bulilima.

### Data collection tools

A pre-tested semi-structured questionnaire was used to collect quantitative data from January 2020 to February 2020 from women who delivered either at the clinic or home in Bulilima district and were captured in health facility registers. The questionnaire was categorised into two sections, i.e., the first section comprises questions on socio-demographic characteristics (age, race, ethnicity, education, marital status, parity). The second section included questions on complications that arose as well as obstetric factors (a type of delivery and pregnancy outcome). It took, on average, 15–20 min to administer the questionnaire. The questionnaire was developed in English and then translated to the local language that is “isi*Ndebele*” which is mainly spoken in the district.

### Data analysis

Data were entered onto EPI DATA Version 3.1 and then exported to Microsoft Excel 2013. The data was then cleaned and checked for completeness in Excel before being further exported to STATA SE Version 13 for analysis. Descriptive statistics were used for demographic characteristics of women utilising traditional medicines. The Fisher’s Exact Test and the Test of Proportions were used to determine whether or not there was a relationship between Traditional Medicine utilisation and the prevalence of maternal complications in women who delivered in Bulilima.

## Results

### Demographics characteristics of women and TM utilisation

Of the 185 targeted respondents, 177 participated in the study giving a response rate of 96%. A significant number of women were having a partner 132 (74.57%), and 139 (78.53%) were Christians, while 110 (62.15%) were unemployed. Only one woman was within the age 50–54, as indicated in Table 1: There was an association between age and TM utilisation evidenced by older women being 13 times more likely to use TM as compared to younger ones. Religion and parity were significantly associated with TM use with those that had more than six children being ten times more likely to utilise Traditional medicine compared to those with one child, and those that were subscribing to traditional religion is almost four times more likely to use TM as compared to those that were Christians. These findings are presented in Table 2.

**Table 1:** Demographic Characteristics of women

Women (*n* = 177)		
	Frequency	%
**Age**		
15–19	38	21.47
20–24	33	18.64
25–29	34	19.21
30–34	26	14.69
35–39	27	15.25
40–44	12	6.78
45–49	6	3.39
50–54	1	0.56
**Marital Status**		
Single	25	14.12
In a relation	59	33.33
Married	60	33.9
Widowed	12	6.78
Divorced	8	4.52
Cohabiting	13	7.34
**Tribe**		
Ndebele	76	42.92
Shona	18	10.17
Kalanga	74	41.81
Tonga	4	2.26
Other	5	2.82
**Place of Delivery**		
Hospital	131	74.01
Home	46	25.99
**Religion**		
Christian	139	78.53
Tradition	25	14.12
None	13	7.34
**Level of Education**		
Primary	48	27.12
O’Level	78	44.07
A’Level	31	17.51
Tertiary	12	6.78
Never attended school	8	4.52
**Employment Status**		
Employed	34	19.21
Self-Employed	33	18.64
Unemployed	110	62.15
**First Child**		
Yes	54	30.5
No	123	69.50
**Number of Children**		
1	54	30.5
2	48	27.1
3	34	19.2
4	24	13.6
5	8	4.50
6	6	3.40
7	1	0.60
8	2	1.10

**Table 2:** Association between demographics and TM use

Traditional Medicines utilisation								
Variable	Didn’t use TM		Used TM		Fisher Exact	MLR-OR	MLR-95% CI	MLR *P*-value
**Age**	Freq	%	Freq	%	0.011*			
15–19^*^	33	25.98	5	10.00		***		
20–24	26	20.47	7	14.00		1.78	0.51–6.25	0.37
25–29	25	19.69	9	18.00		2.38	0.71–7.97	0.16
30–34	18	14.17	8	16.00		2.93	0.84–10.30	0.09
35–39	18	14.17	9	18.00		3.30	0.96–11.35	0.06
40–44	5	3.94	7	14.00		9.24	2.10–40.75	< 0.01*
45–49	2	1.57	4	8.00		13.2	1.90–91.91	< 0.01*
50–54	0.00	0.00	1	1.00		1		
**Marital Status**								
Single^*^	18	14.17	7	14.00	0.853	***		
In a relationship	45	35.43	14	28.00		0.80	0.28–2.31	0.68
Married	42	33.07	18	36.00		1.10	0.39–3.10	0.85
Widowed	7	5.51	5	10.00		1.84	0.43–7.77	0.41
Divorced	6	4.72	2	4.00		0.86	0.14–5.31	0.87
Cohabiting	9	7.09	4	8.00		1.14	0.26–4.95	0.86
**Tribe**								
Ndebele^***^	58	45.67	18	36.00	0.349	***		
Shona	14	11.02	4	8.00		0.92	0.27–3.15	0.90
Kalanga	50	39.37	24	48.00		1.55	0.75–3.17	0.23
Tonga	3	2.36	1	2.00		1.07	0.11–10.97	0.95
Other	2	1.57	3	6.00		4.83	0.75–31.23	0.10
**Religion**								
Christian^***^	104	81.89	35	70.00	0.002*	***		
Traditional	11	8.66	14	28.00		3.78	1.57–9.10	< 0.01*
None	12	9.45	1	2.00		0.25	0.03–1.97	0.19
**Level of education**								
Primary^***^	35	27.56	13	26.00	0.342	***		
O’level	54	42.52	24	48.00		1.20	0.54–2.66	0.66
A’level	23	18.11	8	16.00		0.94	0.34–2.61	0.90
Tertiary	11	8.66	1	2.00		0.24	0.03–2.09	0.20
Never attended school	4	3.15	4	8.00		2.69	0.59–12.37	0.20
**Employment status**								
Employed	22	17.32	12	24.00	0.495	***		
Self Employed	23	18.11	10	20.00		0.80	0.29–2.22	0.66
Unemployed	82	64.57	28	56.00		0.63	0.27–1.43	0.26
**Place of delivery**								
Hospital	99	77.95	32	64.00	0.086	***		
Home	28	22.05	18	36.00		1.99	0.97–4.06	0.06
**First Child**								
Yes	47	37.01	6	12.00	0.001*	***		
No	80	62.99	44	88.00		4.46	1.77–11.24	< 0.01*
**Parity**								
1	48	37.80	6	12.00	0.002*	***		
2–5	75	59.06	39	78.00		4.16	1.64–10.57	< 0.01*
6>	4	3.15	5	10.00		10.00	2.09–47.82	< 0.01*

*** Reference Group

*Significant Results

### Method of delivery and complication

Of the 177 respondents who participated in the study, 35 (20%) had to have an assisted delivery. Of those participating, 51 (29%) reported that they had complications. Furthermore, the prevalence of complications in women who utilised TM was 265 as compared to 30% in those that did not use TM during pregnancy. These findings are presented in Table 3**.**

**Table 3:** Method of Delivery and complications

Variable	Frequency	%
Method of Delivery (*n* = 177)		
Normal	142	80.23
Assisted	35	19.77
Pregnancy complications (*n* = 177)		
Complicated	51	28.81
Did not complicate	126	71.19
Preference in pregnancy complications consultation (*n* = 177)		
TP	11	6.79
HP	119	73.46
Both (HP & TP)	32	19.75
Family		
Views on TM safety (*n* = 177)		
Safe	20	11.30
Not Safe	39	22.03
Don’t Know	118	66.67
Traditional Medicine Utilisation (*n* = 177)		
Yes	50	28.25
No	127	71.75
Proportions of Complications with TM utilisation		
Utilised traditional medicine and had complications	^38^/_127_	29.92
Did not utilise traditional medicines and ha complications	^13^/_50_	26.00

### Maternal complications and utilisation of traditional medicine (TM)

The majority of individuals used traditional medicine and spent much time in labour while there was no significant difference with other types of complications. However, the total number of complications was significantly higher in women who did not use TM as compared to those that utilised TM. These findings are presented in Table 3.

### Pregnancy period, frequency of TM use and types complications

There was no significant association between types of complications and pregnancy period. Also, there was no significant association between frequency of TM use and different kinds of complications among those who utilise TM, as indicated in Table 4.

**Table 4:** Maternal complication and utilisation of Tradition Medicine (TM)

Variable (*n* = 51)	Didn’t use TM		Used TM		Test of proportions *p*-value
Complications	Freq	%	Freq	%	
Prolonged labour	9	23	9	69.23	0.053
Prolonged labour & Vomiting	2	5.26	0	0.00	–
Prolonged labour & pain	2	5.26	0	0.00	–
Amniotic fluid	1	2.63	0	0.00	–
Bleeding	4	10.53	2	15.38	0.864
Breeching	5	13.16	0	0.00	–
Failure to progress	2	5.26	0	0.00	–
Foetal Macrosomia	1	2.63	0	0.00	–
High Blood pressure	0	0.00	1	7.69	–
Stomach pain	6	15.79	0	0.00	–
Stomach and uterus pain	1	2.63	0	0.00	–
Unknown	5	13.16	1	7.69	0.879
Overall comparison for all complications	**38**	**74.5**	**13**	**25.5**	0.004*

*Significant Results

### Complications management among TM users

Women who utilised TM and encountered maternal complications highlighted stress (23.08%) as a predictor of complications, and the majority stated that regular clinic check-ups (30.77%) should be done to overcome complications, as indicated in Table 5.

**Table 5:** Pregnancy Period, Frequency of Traditional Medicine use and complications

**Variable (*n*** **= 13**)	1st Trimester	3rd Trimester	During labour	After labour	**Fisher’s Exact**
**Complication**	Freq (%)	Freq (%)	Freq (%)	Freq (%)	0.476
Bleeding	1 (33.33)	1 (14.29)	0 (0.00)	0 (0.00)	
High blood pressure	0 (0.00)	1 (14.29	0 (0.00)	0 (0.00)	
Prolonged labour	2 (66.67)	5 (71.43)	2 (100.00)	0 (0.00)	
Unknown	0 (0.00)	0 (0.00)	0 (0.00)	1 (100.00)	
**Frequency of TM use**					
	Bleeding	High blood pressure	Prolonged labour	Unknown	
	Freq (%)	Freq (%)	Freq (%)	Freq (%)	
1–5 times	2 (100.00)	1 (100.00)	7 (77.78)	1 (100.00)	1.000
6>	0 (0.00)	0 (0.00)	2 (22.22)	0 (0.00)	

### Reasons and ways to overcome complications

Respondents cited factors that lead to complications and ways of overcoming them. Stress and being overworked were cited as some of the reasons that led to complications during pregnancy. Ways of overcoming these complications were reported to be regular clinic visits as well as utilisation of TM. These findings are presented in Table 6**.**

**Table 6:** Reasons and ways to overcome complications

Variable (***n*** = 13)	Frequency	%
**Reasons for complications**		
Unknown	2	15.38
Stress	3	23.08
Overworking	2	15.38
Poor monitoring from nurses	1	7.69
Age (too old)	1	7.69
Witchcraft	1	7.69
Lack of exercise	1	7.69
Sleeping on the wrong side	1	7.69
Foetal macro soma	1	7.69
**How to overcome complications**		
Regular clinic check-ups	4	30.77
Unknown	3	23.08
Drink a lot of water	1	7.69
Use TM	2	15.38
Consult elders	1	7.69
Should know what to do	1	7.69
Proper sleeping position	1	7.69

## Discussion

The study found that older women and those that have over six children were highly likely to use TM as compared to younger women as well as those that had lesser children. The study also found out that TM utilisation was more common as well in older women as compared to the younger ones. These findings symbolise hat majority of times older women are more cultural and usually subscribe to the cultural norms in their societies as compared to younger women who have mixed beliefs due to cultural pollination [20, 21]. The study further found that those that were subscribing to traditional religion were more likely to use TM as compared to those that did not. These findings are in sync with what is presented in literature where those that value the traditional religion and norms are bound to utilise it for any of their health needs as compared to Christians who usually associated TMs utilisation to witchcraft and acts of demons [22, 23].

The study found that 80% of the women normally delivered regardless of whether or not they utilised traditional medicine. Bin Zakaria (2010) reports high success in women delivering, including those that utilise traditional medicine [24]. Also, there was a significant association between TM use and total proportions of complications among those who utilise TM and those that did not utilise it. Those who utilised TM medicines had lower proportions of women who developed complications as compared to those that did not use TM during pregnancy. These findings contradict with findings presented in a study that was conducted by Siveregi (2019) that reported increased chances of complications in those that utilise TM as compared to those that do not cite higher chances of suffering from complications such as hyper uterine stimulus [25]. This argument is also bolstered by Elkhoudri (2016) in their study that reports that utilisation of traditional medicines presents huge risks for the mother and the baby [26]. Furthermore, studies suggest that due to the lack of regulated doses, usage of TM can lead to hyper uterine stimulus and other complications [27].

The study also found that the prevalence of complications in women who reported to have used TM during pregnancy was lower than the prevalence that was reported among those that did not utilise TM. These findings contradict findings presented by different studies in some different settings that reported that increased up and utilisation of TMs have been increases the chances of women suffering from complications during pregnancy and in some cases leading to the women not disclosing their usage of TMs during pregnancy to the health care providers [28]. There is usually fear for women to declare their utilisation of TMs as they fear most modern health service providers disapprove as there is limited understanding of the efficacy and safety of these TMs [28]. This, however, brings to question the types, nature and dosage of TMs that are used in different country settings where there is a variety of TMs that are used particularly in developing countries [29]. Other authors argue that what usually causes these complications are not necessarily the utilisation of TM, but rather the fact that women seek medication when it is already late when complications have already manifested themselves [30]. In general, there have been reports that TM is effective and works very well [30]. This is particularly true in countries where women seek or use TMs secretively and would only utilise them when there are sensing risks of complications [30–32].

The study revealed stress (23.08%) as a predictor of complications, and a significant number stated that regular clinic check-ups (30.77%) should be done to overcome complications. This finding is in line with what other researchers found; i.e., women with stress are at higher risk of having complications such as pre-term contractions, abortions, bleeding, and low birth weight [33, 34]. It is reported in this study that about 29% of the respondents had complications during their pregnancy. Also, results showed that there is no association between traditional medicine and maternal complications. This finding is contrary to the studies conducted by Mabina et al. (1997), who found out that traditional medicine is associated with foetal distress [35, 36]. Besides, we found out that parity, age is significantly related to different types of complications. Studies concur with our results as they indicated that pre-labour, uterus rupture, pregnancy-induced hypertension, placental complications, and haemorrhage is associated with age and parity [37, 38].

## Limitations

This study was not funded, and as such, there could have been a need for a substantial cohort to make meaningful inferences. However, as part of the more significant project that authors are involved in, we intend to explore the chemical components that are found in the different types of the TMs that are typically used during pregnancy by women.

## Conclusion

In conclusion, older women, those with more than six children, those that subscribe to traditional religion were more likely to use TMs. This study also found that overall utilisation of TM could reduce the chances of complications as shown by the lower proportion of women who utilised TM and developed complications compared to those that did not utilise TM. Despite this finding, some studies have found that traditional medicine is not totally safe, with a significant number of women complicating after taking TM. However, on the contrary, TM has been reported by several studies to be of importance in health systems, including in the management of pregnancies. Adopting a cautious approach in utilising TM, conducting more research (which is currently ongoing) on the different types used in different contexts and isolation the active ingredients, assessing frequencies of TM intakes, could provide more insight and information to create inventories as TM could provide complementary solutions to the management of pregnancies, particularly in low-income countries where the majority struggle to afford costs associated with maternal services offered in modern health systems.

## Data Availability

Not Applicable.
